# Coronary artery bypass surgery in a patient with Kartagener syndrome: a case report and literature review

**DOI:** 10.1186/1749-8090-5-68

**Published:** 2010-08-26

**Authors:** Ioannis Bougioukas, Dimitrios Mikroulis, Bernhard Danner, Lukman Lawal, Savvas Eleftheriadis, George Bougioukas, Vassilios Didilis

**Affiliations:** 1 Dpt. of Cardiothoracic Surgery, University Hospital Alexandroupolis, 68100, Greece; 2 Dpt of Cardiovascular and Thoracic Surgery, University Hospital Goettingen, Germany; 3 Dpt. of Anesthesiology. University Hospital Alexandroupolis, 68100, Greece

## Abstract

Kartagener syndrome consists of congenital bronchiectasis, sinusitis, and total situs inversus in half of the patients. A patient diagnosed with Kartagener syndrome was reffered to our department due to 3-vessel coronary disease. An off-pump coronary artery bypass operation was performed using both internal thoracic arteries and a saphenous vein graft. We performed a literature review for cases with Kartagener syndrome, coronary surgery and dextrocardia. Although a few cases of dextrocardia were found in the literature, no case of Kartagener syndrome was mentioned.

## Introduction

In 1606 Hieronymous Fabricious described situs inversus, while in 1643 Marco Severino described dextrocardia [1]. Situs inversus is a rare congenital disorder with an incidence of 1:10000, in which the major visceral organs are reversed from left to right in a mirror image of the normal condition [2]. Kartagener syndrome consists of congenital bronchiectasis, dextrocardia and sinusitis [2].

A patient with Kartagener's syndrome and three-vessel coronary disease was referred to our department for bypass surgery. We searched the literature about the Kartagener's syndrome in order to find references about the choice of conduits and the position of the surgeon in patients with mirror-image appearance of the heart. Several cases of surgical coronary revascularization in patients with dextrocardia have been reported in the literature, but no case was referred as Kartagener's syndrome. We report a case of a patient with Kartagener's syndrome with total situs inversus, bronchiectasis, chronic respiratory disease and three-vessel coronary disease, being treated in our institute with coronary surgery using both internal thoracic arteries. To the best of our knowledge this is the first report of coronary surgery in a patient with Kartagener syndrome.

## Case Report and Review

**Figure 1:**
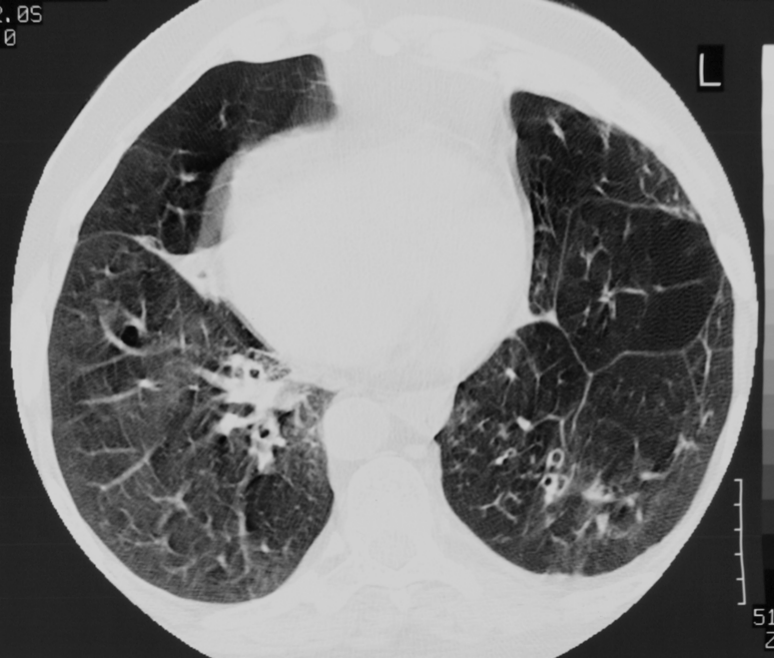
**CT scan of the thorax showing dextrocardia and bronchiectasis of the lungs**.

A 56 year-old Caucasian male patient was admitted to our department for scheduled coronary artery bypass due to three-vessel coronary disease. The patient was already diagnosed as Kartagener syndrome with total situs inversus and azoospermia (patient had no children). A CT scan of the thorax showed bronchiectasis of the lungs and dextrocardia (fig. 1). The coronary angiography was performed without particular difficulties and revealed a proximal stenosis of 90% in the left anterior descending artery (LAD), a proximal stenosis of 90% in the circumflex artery and a stenosis of 99% between the proximal and middle part of the right coronary artery. The ejection fraction was normal and the aortic valve was competent. A spirometry was performed which revealed a reduction of the Forced Expiratory Volume, with a FEV1 of 1.44 L (40.6% of predicted value) and a reduction of the Forced Vital Capacity, with a FVC of 1.80 L (38.7% of the predicted value). Due to the patient's severe pulmonary disease an off-pump operation was decided.

The chest was entered through a median sternotomy, with the surgeon standing on the left side of the patient. The heart had an exact mirror image of a normally positioned heart and showed a good contractility. Both internal mammary arteries (IMAs) and a saphenous vein graft (SVG) were harvested. The LAD was opened and grafted with the left internal mammary artery (LIMA). Then the first obtuse marginal branch of the circumflex artery was grafted with right internal mammary artery (RIMA). Finally, the posterior descending artery (PDA) was grafted with the saphenous vein graft. The proximal anastomosis of the vein graft was then performed on the ascending aorta. After haemostasis, the chest was closed in routine fashion. The patient was extubated six hours later and remained in the Intensive Care Unit for three days due to his respiratory disease and increased volume of secretions. He was discharged from the hospital on the 10^th ^postoperative day.

## Discussion

Kartagener's syndrome is characterized by the triad of bronchiectasis, sinusitis and situs inversus, and is also combined with abnormalities of the cilia of the respiratory epithelium. Some male patients with Katagener's syndrome also have sterility due to dyskinesia of the spermatozoa [2].

Total situs inversus is a rare condition which does not preclude long-term survival. Patients with dextrocardia and coronary disease may present for coronary bypass surgery. The mirror-image site of the heart and the great vessels does not impose a problem for carrying out a normal coronary artery bypass grafting operation, as it can be seen in the literature.

Saad et al reviewed the literature for coronary surgery in patients with dextrocardia, dealing with the position of the surgeon [3]. We reviewed the literature in order to ascertain the conduit choice of each surgeon, especially concerning grafting of the left anterior descending artery (Table 1).

**Table 1:** Literature review of coronary surgery in dextrocardia patients.

Authors	Operation	Grafts used	Comments
Grey, Cooley (1981)[9]	1. CABGx5 2. CABGx2 3. CABGx2	SVGs	
Irvin (1982)[10]	CABGx3	SVGs	
Yamaguchi (1990)[11]			
Astudillo (1993)[12]	CABG	SVG	
Nomoto (1997)[13]	CABG	SVG	LM disease
Seddio (1999)[4]	1. CABGx1 2. CABGx1	RIMA LIMA	RIMA to LAD Free LIMA to LAD
Wong, Chong (1999)[16]	CABGx3	RIMA, SVGs	
Totaro (2001)[17]	CABGx3	RIMA, SVGs	
Tabry (2001)[5]	CABGx4	RIMA, free LIMA, SVG	RIMA to free LIMA to D1 and LAD, RIMA to OM1 to OM2, SVG to PDA
Naik (2002)[18]	CABGx2	RIMA, SVG	
Erdil (2002)[19]	CABGx2	RIMA, SVG	
Stamou (2003)[20]	CABGx2	RIMA, SVG	
Chui, Sarkar (2003)[21]	CABGx2	RIMA, RA	
Bonde, Campalani (2003)[15]	CABGx2	RIMA, SVG	
Bonanomi (2004)[22]	CABGx2	RIMA, SVG	
Abdullah, Mazalan (2004)[23]	CABGx3	SVGs	
Kuwata (2004)[6]	CABGx5	Both IMAs Both RAs	In situ LIMA to LAD
Poncelet (2006)[24]	CABGx3	Both IMAs GEA	
Ennker (2006)[25]	CABGx2	RIMA	
Karimi (2007)[26]	1. CABGx3 2. CABGx4	RIMA, SVGs RIMA, SVGs	
Pego-Fernadez (2007)[27]	CABGx5	RIMA, SVGs	
Saadi (2007)[28]	CABGx3	RIMA, SVGs	
Chakravarthy (2008)[7]	1. CABG 2. CABG	LIMA, RA, SVG RIMA, SVG	In situ LIMA to LAD
Saad (2009)[3]	CABGx3	RIMA,SVGs	
Yamashiro (2009)[8]	CABGx4	Both IMAs, RA	RIMA to LAD, LIMA to OM1, LIMA to RA to OM2 to PDA

Most of the authors preferred to graft the LAD with the right internal mammary artery, as the mirror-image appearance of the heart offers the convenience of using this arterial graft.

Seedio et al. reported a series of two patients [4]. In one case they used LIMA as a free graft to graft the LAD. Tabry et al. anastomosed the free LIMA to the RIMA and then they grafted the LIMA to the first diagonal branch and the LAD [5]. Kuwata et al. harvested both internal mammary arteries and both radial arteries, skeletonized the LIMA and managed to use it in-situ to graft the LAD [6]. Chakravarthy et al. reported two cases [7]. In the first case, they used LIMA in-situ to graft the LAD, whereas in the second case they used the RIMA. Yamashiro et al. used both IMAs and the radial artery, which was anastomosed to the LIMA and then to the second obtuse marginal branch (OM2) and PDA in a sequential manner [8]. RIMA was anastomosed to the LAD and LIMA grafted the OM1 branch. In older reports (Grey and Cooley, Irvin, Yamaguchi, Astudillo, Nomoto) saphenous vein grafts were exclusively used [9-13].

In our case the use of the left internal mammary artery to graft the left anterior descending artery was feasible, as the stenosis of the vessel was proximal and the length of the arterial conduit imposed no technical difficulty. We preferred the use of the LIMA to the LAD as the literature has strongly proven the excellent results of this anastomosis [14]. RIMA was skeletonized and used to graft the obtuse marginal branch of the circumflex artery. Finally, performing the operation "off-pump" did not constitute a problem in our case, as the patient was haemodynamically stable throughout the procedure allowing us to have access to all coronary vessels, without the need of conversion to "on-pump" operation, as occurred in the case of Bonde and Campalani [15]. The use of cardiopulmonary bypass was omitted in our patient because of his poor respiratory function.

## Conclusion

Situs inversus with mirror-image of the heart is a rare condition, which eventually every cardiac surgeon might have to deal with. The position of the surgeon depends mainly on the surgeon's choice. The use of the RIMA seems to be the easier way to graft the LAD, but when the lesion of the LAD is proximal LIMA can also be used to graft the LAD. In patients with Kartagener's syndrome and severe respiratory disease, off-pump bypass grafting could be performed.

## Abbreviations

CT: Computed Tomography; FEV1: Forced Expiratory Volume in 1 second; FVC: Forced Vital Capacity; LAD: Left Anterior Descending artery; OM: Obtuse Marginal branch; PDA: Posterior Descending Artery; LIMA: Left Internal Mammary Artery; RIMA: Right Internal Mammary Artery; SVG: Saphenous Vein Graft.

## Competing interests

The authors declare that they have no competing interests.

## Authors' contributions

Author's contributions: IB was the author. LL, BD and DM contributed to literature research. VD was the surgeon and supervisor. SE was the anesthetist. GB made corrections and consultation. All authors read and approved the final manuscript.

## References

[B1] Cleveland M Situs in versus viscerum: anatomic study Arch surg 1926 13 343

[B2] Leigh M Pittman J Carson J Ferkol T Dell S Davis S Knowles M Zariwala M Clinical and genetic aspects of primary ciliary dyskinesia/Kartagener syndrome Genet med 2009 11 7 473 487 10.1097/GIM.0b013e3181a53562 19606528 PMC3739704

[B3] Saad R Badr A Goodwinm A Dunning J Should you stand on the left or the right of a patient with dextrocardia who needs coronary surgery? Interact CardioVasc Thorac Surg 2009 9 698 702 10.1510/icvts.2009.216317 19638356

[B4] Seddio F Colagrande L Pellegrino A De Paulis R Bassano C Chiariello L Myocardial revascularization in dextrocardia with situs inversus G Ital Cardiol 1999 29 10 1222 6 10546139

[B5] Tabry I Calabrese J Zammar H Abou-Kasem K Akeilan H Gharbieh N Zinati H Noureddine W el-Hout A Tayah M Khalidy L Yaghi M Case report: off-pump total myocardial revascularization for dextrocardia and situs inversus Heart Surg Forum 2001 4 3 251 3 11673147

[B6] Kuwata T Ueda T Sakaguchi H Nagasaka S Taniguchi S Off-pump quintuple coronary artery bypass grafting for situs inversus totalis Jpn J Thorac Cardiovasc Surg 2004 52 10 473 5 10.1007/s11748-004-0143-7 15552972

[B7] Chakravarthy M Jawali V Nijagal D Off-Pump Coronary Artery Bypass Surgery in Dextrocardia: A Report of Two Cases Ann Thorac Cardiovasc Surg 2008 14 3 187 191 18577901

[B8] Yamashiro S Iha K Akasaki M Toru Uezu Ryo Ikemura Isao Nishijima Emergency off-pump complete arterial revascularization in a patient with dextrocardia Gen Thorac Cardiovasc Surg 2009 57 625 628 10.1007/s11748-009-0446-9 19908120

[B9] Grey D Cooley D Dextrocardia with situs inversus totalis: Cardiovascular surgery in three patients with concomitant coronary artery disease Cardiovascular Diseases, Bulletin of the Texas Heart Institute 1981 8 4 PMC287993 15216178

[B10] Irvin R Ballenger J Coronary artery bypass surgery in a patient with situs inversus Chest 1982 81 380 381 10.1378/chest.81.3.380 6976885

[B11] Yamaguchi T Kikuchi S Doi H Watanabe A Ebuoka M Coronary artery bypass in dextrocardia with situs inversus totalis--a case report Nippon Kyobu Geka Gakkai Zasshi 1990 38 9 1538 42 2246545

[B12] Astudillo R Escudero X Farell J Ariza H González Carmona VM Tello R Atherosclerotic ischemic cardiopathy in patients with dextrocardia in situs viscerum inversus Arch Inst Cardiol Mex 1993 63 2 123 6 8503712

[B13] Nomoto T Ueda Y Ogino H Sugita T Morioka K Matsubayashi K Emergent coronary artery bypass grafting in a patient with mirror-image dextrocardia Kyobu Geka 1997 50 9 785 8 9259142

[B14] Mack M Osborne J Shennib H Arterial graft patency in coronary bypass grafting: what do we really know? Ann Thorac Surg 1998 66 1055 1059 10.1016/S0003-4975(98)00815-7 9769002

[B15] Bonde P Campalani G Myocardial revascularization for situs inversus totalis and dextrocardia Interactive Cardiovascular and Thoracic Surgery 2003 2 486 488 10.1016/S1569-9293(03)00125-7 17670102

[B16] Wong PS Chong CL Multiple coronary artery bypass grafting in dextrocardia: case report Med J Malaysia 1999 54 4 514 6 11072472

[B17] Totaro P Coletti G Lettieri C Pepi P Minzioni G Coronary artery bypass grafts in a patient with isolated cardiac dextroversion Ital Heart J 2001 2 5 394 6 11392646

[B18] Naik MJ Chua YL Ding ZP Lau KW Coronary artery bypass grafts in situs inversus J Cardiovasc Surg (Torino) 2002 43 2 181 4 11887051

[B19] Erdil N Cetin L Sener E Ufuk D Cemal S Situs Inversus and Coronary Artery Disease Asian Cardiovasc Thorac Ann 2002 10 53 4 12079972 10.1177/021849230201000113

[B20] Stamou S Bafi A Kapetanakis E Lowery R Pfister A Dullum M Boyce S Corso P Beating Heart Surgery in a Patient with Dextrocardia and Complete Situs Inversus J Card Surg 2003 18 170 172 10.1046/j.1540-8191.2003.02008.x 12757348

[B21] Chui W Sarkar P Coronary artery bypass grafting in dextrocardia with situs inversus totalis J Cardiovasc Surg (Torino) 2003 44 5 617 9 14735049

[B22] Bonanomi G Kostov D Zenati M Emergent off-pump complete myocardial revascularization in dextrocardia J Cardiovasc Surg (Torino) 2004 45 1 31 3 15041933

[B23] Abdullah F Mazalan S Off-pump coronary artery bypass grafting in a high-risk dextrocardia patient: a case report Heart Surg Forum 2004 7 E186 E188 10.1532/HSF98.20033016 15262598

[B24] Poncelet A Dion R Lengele B Noirhomme P Complete arterial revascularization in coronary artery bypass grafting in a patient with solitus inversus totalis J Cardiovasc Surg (Torino) 2006 47 4 477 9 16953169

[B25] Ennker I Pietrowski D Ennker J Off-pump myocardial revascularization in an octogenarian patient with dextrocardia and situs inversus Cardiovascular Journal Of South Africa 2006 17 5 257 258 17117232

[B26] Karimi A SalehiOmran A Ahmadi H Yazdanifard P Total myocardial revascularization for situs inversus totalis with dextrocardia: a case report Journal of Medical Case Reports 2007 1 18 10.1186/1752-1947-1-18 17480236 PMC1884161

[B27] Pego-Fernandes PM de Serro-Azul JB Matheus F Maehara BS Myocardial revascularization in a patient with situs inversus totalis Arq Bras Cardiol 2007 88 5 e103 6 10.1590/S0066-782X2007000500021 17589612

[B28] Saadi E Dussin L Nicolao A Zago A Coronary artery bypass grafting in a patient with situs inversus totalis and dextrocardia Rev Bras Cir Cardiovasc 2007 22 3 346 348 10.1590/S0102-76382007000300012 18157421

